# GTP Binding Is Necessary for the Activation of a Toxic Mutant Isoform of the Essential GTPase ObgE

**DOI:** 10.3390/ijms21010016

**Published:** 2019-12-18

**Authors:** Liselot Dewachter, Babette Deckers, Ella Martin, Pauline Herpels, Sotirios Gkekas, Wim Versées, Natalie Verstraeten, Maarten Fauvart, Jan Michiels

**Affiliations:** 1 Centre of Microbial and Plant Genetics, KU Leuven—University of Leuven, Kasteelpark Arenberg 20 Box 2460, B-3001 Leuven, Belgium; 2 VIB-KU Leuven Center for Microbiology, Kasteelpark Arenberg 20 Box 2460, B-3001 Leuven, Belgium; 3 Structural Biology Brussels, Vrije Universiteit Brussel, Pleinlaan 2, B-1050 Brussels, Belgium; 4 VIB-VUB Center for Structural Biology, Pleinlaan 2, B-1050 Brussels, Belgium; 5 Imec, Kapeldreef 75, B-3001 Leuven, Belgium

**Keywords:** Obg, ObgE, GTPase, GTP binding, *Escherichia coli*

## Abstract

Even though the Obg protein is essential for bacterial viability, the cellular functions of this universally conserved GTPase remain enigmatic. Moreover, the influence of GTP and GDP binding on the activity of this protein is largely unknown. Previously, we identified a mutant isoform of ObgE (the Obg protein of *Escherichia coli*) that triggers cell death. In this research we explore the biochemical requirements for the toxic effect of this mutant ObgE* isoform, using cell death as a readily accessible read-out for protein activity. Both the absence of the N-terminal domain and a decreased GTP binding affinity neutralize ObgE*-mediated toxicity. Moreover, a deletion in the region that connects the N-terminal domain to the G domain likewise abolishes toxicity. Taken together, these data indicate that GTP binding by ObgE* triggers a conformational change that is transmitted to the N-terminal domain to confer toxicity. We therefore conclude that ObgE*–GTP, but not ObgE*–GDP, is the active form of ObgE* that is detrimental to cell viability. Based on these data, we speculate that also for wild-type ObgE, GTP binding triggers conformational changes that affect the N-terminal domain and thereby control ObgE function.

## 1. Introduction

Obg proteins belong to the TRAFAC (translation factor) class of P-loop GTPases that typically switch between a GTP- and a GDP-bound state by GTP hydrolysis or nucleotide exchange [1,2]. Because the GTP hydrolysis rate of Obg is low and nucleotide exchange occurs very fast, the nucleotide binding state of Obg is thought to be mainly controlled by the intracellular ratio of GTP and GDP [2,3,4,5]. Interestingly, Obg also binds ppGpp with physiologically relevant affinity [6,7]. 

Obg GTPases generally consist of three distinct domains. The N-terminal Obg domain is widely conserved and is essential for Obg function [2,8]. It consists of six left-handed type II helices and an eight-stranded β-barrel that contacts the G domain [8,9,10]. The Obg domain most likely mediates protein–protein interactions with effector molecules [8,11]. In *Escherichia coli* Obg (ObgE), this domain spans from amino acid residue 1 until 157 [9]. Nucleotide binding and hydrolysis is performed by the G domain. This domain consists of a six-stranded β-sheet and five α-helices and undergoes conformational changes in response to nucleotide binding [1,2,8,9,10]. These conformational changes are thought to be transmitted to the N terminus through a flexible linker region between the G and N domains [8,12]. In *E. coli*, the G domain contains amino acid residues 158 to 340 [9]. The final 50 residues of ObgE make up the C-terminal domain [9]. This domain is not well conserved among different species and is therefore thought to mediate species-specific Obg functions [2]. In *E. coli*, the C-terminus of ObgE is intrinsically disordered [9].

The Obg protein is widely-conserved and essential for viability in all bacteria tested to date [2]. It plays a role in a variety of important cellular processes, such as ribosome assembly, the stringent response, morphological development, and antibiotic tolerance [2,6,13,14,15]. Its essential function, however, is thought to stem from its involvement in cell cycle progression, where it has been shown to influence the initiation of DNA replication, chromosome segregation, and cell division [2,16,17,18,19,20]. None of these roles of Obg have been characterized mechanistically, leaving the exact molecular functions of Obg enigmatic. Moreover, although Obg is a GTPase with distinct structural domains, the effect of nucleotide binding on protein function remains largely unexplored and it is unclear which protein domains contribute to specific functions.

Here, we set out to help bridge the gap between the *in vitro* structural and biochemical data on the ObgE protein and its *in vivo* behavior. More specifically, we investigate the influence of different protein domains and nucleotide binding on the toxic effect of a mutant isoform of ObgE, called ObgE*. ObgE* contains a K268I amino acid substitution, which is located in the G domain of the protein [19,21]. This mutant protein causes a severe loss of viability in *E. coli*, thereby providing an easy way to assess one aspect of ObgE activity [19,21]. Our results indicate that, besides the mutated G domain, also the N-terminal domain of ObgE* is necessary to confer toxicity. Moreover, the integrity of the linker region between both domains appears to be essential. Additionally, we observed that mutations that lead to a decreased GTP binding affinity rescue the toxic effect of ObgE*. These data suggest that the conformational change that occurs upon GTP binding needs to be correctly transmitted to the N-terminal Obg domain in order for ObgE* to exert its toxic effect. Our findings thus indicate that the toxic activity of ObgE* *in vivo* depends on its nucleotide binding state and that information on this nucleotide binding state is transmitted to the N-terminal Obg domain to influence protein activity. 

## 2. Results

### 2.1. The N-Terminal and G Domains of ObgE* Are Necessary for Toxicity

**Figure 1:**
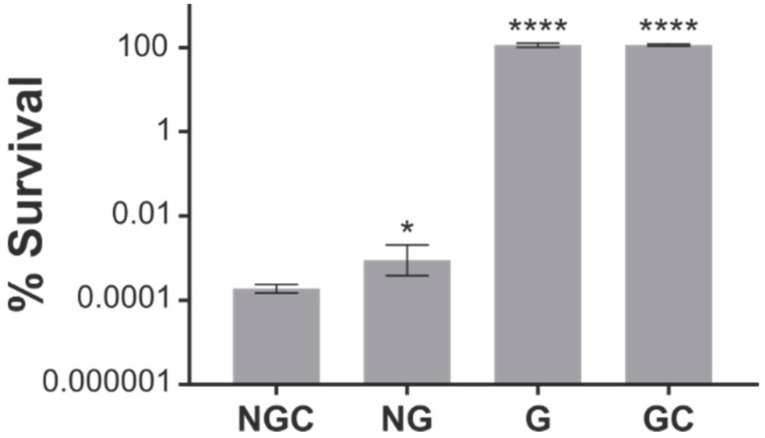
ObgE*-mediated toxicity requires both the N-terminal and G domains of ObgE*. Survival was determined by dividing the number of CFUs per mL obtained after expression of ObgE* domain mutants by the number of CFUs per mL after expression of the corresponding wild-type ObgE domain mutant (NGC, full-length protein; NG, the N-terminal and G domains; G, the G domain; GC, the G and C-terminal domains). Data are represented as averages ± SEM, *n* ≥ 3 (* *p* < 0.05, **** *p* < 0.0001, compared to NGC).

The K268I substitution is responsible for the dominant negative effect of ObgE*. ObgE* causes cell death in *E. coli* even at very low expression levels despite the presence of the chromosomally-expressed wild-type ObgE protein [21]. The K268I mutation is situated in the G domain of the protein. We therefore wondered whether this mutated G domain is sufficient to lower viability. To answer this question, expression vectors were constructed that encode only the G domain (G), the N-terminal and G domains (NG), or the G and C-terminal domains (GC) of both ObgE and ObgE*. Survival upon expression of the ObgE* constructs was measured and compared to survival in the presence of the full-length protein (NGC). As is clear from Figure 1, ObgE* toxicity is completely abolished when only the mutated G domain is expressed. The G domain by itself is thus insufficient to lower viability. Likewise, the combination of the mutated G domain with the C-terminal domain has no negative effect on survival. When the N-terminal domain and G domain are combined, however, survival strongly decreases. Interplay between the N-terminal Obg domain and the GTPase domain is thus necessary for ObgE* to exert its toxic effect. Although the C-terminal domain is not necessary for toxicity, its presence can further decrease survival ± five-fold. 

### 2.2. ObgE* Toxicity Is Influenced by Its Nucleotide Binding State

ObgE is capable of binding GTP, GDP, and ppGpp and can also weakly catalyze the hydrolysis of GTP [2,3]. By using an array of amino acid substitutions that alter ObgE’s nucleotide binding affinities [7], we investigated the effect of the nucleotide binding state of ObgE* on toxicity. All amino acid substitutions used are listed in Table 1, together with their effect on the affinity of ObgE for GDP, the non-hydrolysable GTP analog GTPγS and ppGpp. The location of these amino acid residues in the ObgE protein is shown in Figure S1. The genetic changes underlying these amino acid alterations were introduced in obgE* and their effect on toxicity was determined. 

**Figure 2:**
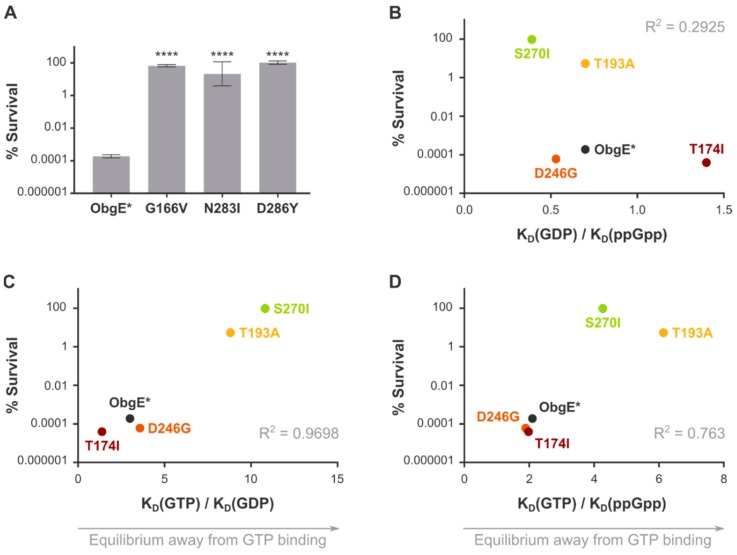
ObgE* needs to bind GTP to confer toxicity. (**A**) Survival was determined upon expression of ObgE* and several ObgE* mutants that are unable to bind nucleotides. Survival is calculated by dividing the number of CFUs per mL; obtained after expression of ObgE* or ObgE* mutants by the number of CFUs per mL obtained after expression of wild-type ObgE. Data are represented as averages ± SEM, *n* ≥ 3 (**** *p* < 0.0001, compared to ObgE*). (**B**) Survival upon expression of ObgE* mutants with altered nucleotide binding affinity was measured and is shown as a function of the K_D_(GDP)/K_D_(ppGpp) ratio of the ObgE mutants. The higher this value, the more the equilibrium is shifted away from GDP binding towards ppGpp binding. (**C**) Survival upon expression of ObgE* mutants with altered nucleotide binding affinity was measured and is shown as a function of the K_D_(GTP)/K_D_(GDP) ratio of the ObgE mutants. The higher this value, the more the equilibrium is shifted away from GTP binding towards GDP binding. (**D**) Survival upon expression of ObgE* mutants with altered nucleotide binding affinity was measured and is shown as a function of the K_D_(GTP)/K_D_(ppGpp) ratio of the ObgE mutants. The higher this value, the more the equilibrium is shifted away from GTP binding towards ppGpp binding. R², squared Pearson correlation coefficient.

First, as is shown in Figure 2A, the introduction of amino acid substitutions that abolish nucleotide binding (G166V, N283I, and D286Y) completely neutralizes ObgE*-mediated toxicity. We therefore conclude that nucleotide binding is a prerequisite for ObgE*’s toxic activity.

After establishing that nucleotide binding is necessary for ObgE*-mediated toxicity, we set out to identify which nucleotide-binding state of ObgE* is responsible for lowering viability. Therefore, the effect of the T174I, T193A, D246G, and S270I amino acid substitutions on ObgE*-mediated toxicity was determined. These amino acid substitutions influence binding of GTP, GDP, and ppGpp in different ways and can therefore be used to determine which nucleotide ObgE* needs to bind to exert its toxic effect. As shown in Supplementary Figure S2, ObgE* toxicity is unaltered in a ΔrelA ΔspoT strain that is unable to synthesize (p)ppGpp, thereby conclusively eliminating any role for (p)ppGpp binding in ObgE* toxicity. Moreover, there is no clear correlation between viability and the individual binding affinities for GDP, GTP, or ppGpp (Figure S2). However, rather than looking at the absolute effect of these mutations on the affinity for each nucleotide separately, it should be kept in mind that in an *in vivo* setting the overall average nucleotide-bound state of ObgE is determined by the ratio of its binding affinities for the different nucleotides. For example, if the GTP binding affinity of a certain ObgE mutant lowers two-fold, but its affinity for GDP is decreased by a factor 10, we expect the *in vivo* ratio of GTP- versus GDP-bound protein to be shifted towards the GTP-bound state. We therefore determined changes in the equilibrium binding state of ObgE mutants by calculating the ratio of individual binding affinities (Table 1). No correlation between toxicity and the ratio of the equilibrium dissociation constants (K_D_)(GDP)/K_D_(ppGpp) can be found (Figure 2B). However, when looking at the K_D_(GTP)/K_D_(GDP) and the K_D_(GTP)/K_D_(ppGpp) ratios, clear correlations emerge (Figure 2C,D). In both cases, the higher these ratios are—and thus the more the equilibrium is shifted away from the GTP-bound state—the higher the viability upon ObgE* expression is. We therefore conclude that ObgE* confers toxicity mainly in its GTP-bound form.

An alternative explanation for the absence of toxicity in ObgE* mutants with abolished or altered nucleotide binding is that the selected mutations lead to decreased expression and/or increased degradation. A Western blot analysis of these mutants shows that this is not the case (Figure S3A). Alternatively, the mutations present could prevent correct folding of the ObgE* protein and therefore no longer lead to a decrease in viability. To exclude this possibility, we took advantage of ObgE being essential for viability [2,3]. We assessed whether the non-toxic obgE* alleles (obgE*_G166V_, obgE*_S270I_, obgE*_N283I_, and obgE*_D286Y_) could function as the sole copy of ObgE in *E. coli*. While expression of these mutant alleles was induced from a plasmid, the chromosomal wild-type *obgE* gene was deleted. With the exception of obgE*_G166V_, all non-toxic obgE* alleles could still support viability and are thus correctly folded (Figure S3B). The lack of toxicity upon expression of these mutants can thus be attributed to their changed nucleotide binding affinities. 

### 2.3. Altering the Linker Region between the N-Terminal and G Domains of ObgE* Neutralizes Toxicity

**Figure 3:**
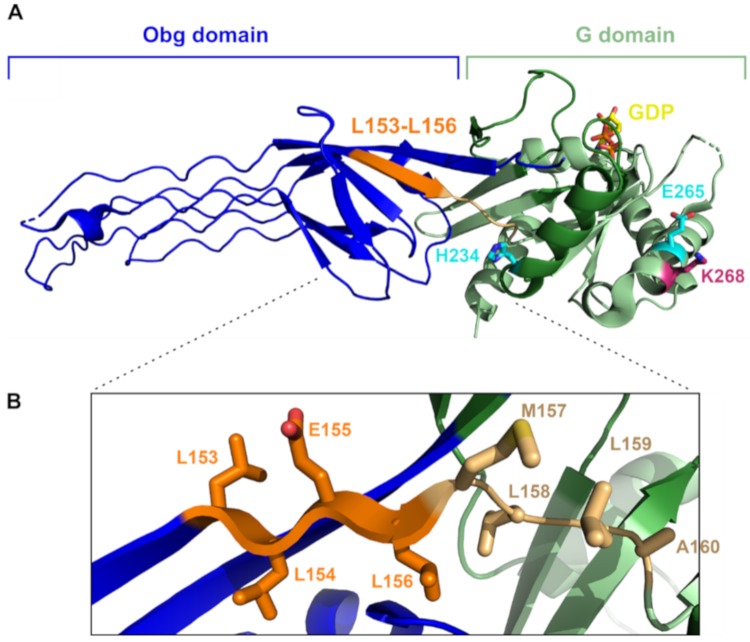
Crystal structure of ObgE showing the position of amino acid changes that neutralize ObgE*-mediated toxicity. (**A**) Schematic representation of the crystal structure of ObgE lacking the C-terminal domain. The N-terminal Obg domain is shown in blue, while the G domain of the protein is highlighted in green. The switch I and II regions are colored in a darker shade of green and the bound GDP molecule is shown in yellow. The K268 residue that is mutated in ObgE* is indicated in pink. The four amino acids that are deleted in many spontaneous compensatory mutants are highlighted in orange (LLEL, positions 153 to 156). Some of the other compensatory mutations are shown in cyan (H234 and E265). (**B**) A close-up view of the region connecting the Obg and G domains. The four amino acids that are deleted in many spontaneous compensatory mutants (LLEL, positions 153 to 156) are directly followed by a rather similar motif in the linker region that connects the Obg and G domains (MLLA). We hypothesize that the MLLA stretch of the linker region can replace the deleted LLEL residues of the last β-strand in the β-barrel subdomain, which otherwise would most likely be deleterious to the fold. If true, the linker between the Obg and G domain is shortened in the Δ153–156 mutant, which could affect the movement of both domains relative to each other.

To identify additional genetic changes in obgE* capable of neutralizing toxicity, we generated spontaneous compensatory mutations. In order to do so, we induced ObgE*-Venus expression on plate and scored colony development. The fluorescent fusion protein ObgE*-Venus retains full toxicity [19] and was used to enable a counter selection against non-fluorescent mutants in which ObgE* expression is abolished. Yellow fluorescent colonies correspond to mutants that express full-length ObgE* but are still able to grow. These colonies were selected and their ObgE*-Venus-encoding plasmids were isolated and transformed into a new *E. coli* strain. In this fresh background, the absence of toxicity upon induction of ObgE* was confirmed to eliminate strains that are resistant to ObgE* expression due to chromosomal mutations. This way, we selected plasmids that carry compensatory mutations in obgE*. Initially, 20 such plasmids—originating from 10 independent overnight cultures—were isolated and sequenced. No single-nucleotide polymorphisms were detected in the selected plasmids. All plasmids did, however, contain the exact same 12-bp deletion in the obgE* gene. This in-frame deletion results in the elimination of amino acid residues LLEL spanning position 153 to 156 in the ObgE* protein. The positions of the deleted residues are highlighted in Figure 3.

It is striking that all 20 obgE* alleles contain the exact same 12-bp deletion and that no other mutations were selected, especially since several ObgE* mutant proteins were identified that no longer cause toxicity (Figure 2). A possible explanation for why only this 12-bp deletion was selected is that it arises more easily than other types of mutations. The detected deletion in obgE* occurs in a region with repeated CTG codons. Such repetitive sequences are extremely sensitive to replication errors and are often deleted during replication [22]. Deletions in this region might therefore occur more frequently, which could explain why only this non-toxic allele was isolated. To circumvent this issue, we altered the repeated CTG codons in this region to synonymous but non-identical codons.

### 2.4. Spontaneous Mutations in ObgE* That Neutralize Toxicity Also Decrease GTP Binding

Starting from the obgE*-*venus* construct with unique codons for amino acids 153 to 156, we again isolated spontaneous compensatory mutations in obgE*. Out of the nine mutant obgE* alleles sequenced, five carried a reversion of the original K268I mutation (Table 2), indicating that the easiest and/or best way to counteract ObgE*-mediated toxicity is to eliminate the causative mutation and revert to wild-type ObgE. Four additional mutations that can likewise neutralize toxicity were identified (Table 2). One of them is again an in-frame deletion in a highly repetitive genetic region in the G domain, highlighting the sensitivity of such repetitive regions to replication errors. Two other mutations affect the glutamic acid residue at position 265 and change it to aspartic acid or lysine. Finally, also the substitution of histidine by arginine at position 234 can counteract ObgE* toxicity (Figure 3A).

As was done for the ObgE mutants with known nucleotide binding affinities, we confirmed expression levels and proper folding of the obgE* alleles selected here by assessing their capability to support growth (Figure S3). Perhaps not surprisingly, the ObgE*_Δ153–156LLEL_ protein is incapable of supporting viability and ObgE*_Δ298–299ES_ allows only limited growth. We can therefore not be sure that these mutant proteins are properly folded. The other mutants are fully capable of supporting viability and thus fold correctly.

To assess the influence of a selection of these mutations on nucleotide binding, we purified ObgE_Δ153–156_, ObgE_H234R_, and ObgE_E265K_. SDS-PAGE gels of the purified proteins are shown in Figure S4. We then performed isothermal titration calorimetry and determined equilibrium binding constants of these mutant proteins (Figure S5). Although the K_D_(GDP)/K_D_(ppGpp) value is more or less constant in these mutants, the equilibrium is clearly shifted towards lower GTP binding in all of them (Table 3). These data thus indicate that the H234R and E265K substitutions eliminate toxicity by lowering GTP binding. These results therefore confirm our previous findings from rationally selected ObgE mutants that show that GTP binding is essential for ObgE*-mediated toxicity (Figure 2). Moreover, the ObgE_Δ153–156_ mutant protein shows a moderate decrease in GTP binding which is expected to contribute to neutralizing ObgE* toxicity. However, since the effect of the Δ153–156 deletion on the nucleotide binding ratios is rather small, we suspect other factors to be at play as well (see Discussion).

## 3. Discussion

In this study we present evidence that implies that ObgE*-mediated cell death is triggered by the GTP-bound version of the protein. Since the G domain itself is insufficient to cause toxicity, we postulate that GTP binding by ObgE* leads to changes in the N-terminal domain that trigger cell death. 

Although the mutated residue of ObgE*, K268, is located in the G domain, expression of this mutated domain is not sufficient to cause cell death. Rather, a combination of the N-terminal and G domains is necessary to confer toxicity (Figure 1). It therefore follows that interaction between the mutated G domain and the N-terminus leads to a loss of viability by ObgE*. The C-terminal domain, on the other hand, is not essential for ObgE*-mediated cell death, although its presence can increase toxicity by a factor 5. The ObgE C-terminus is intrinsically disordered [9], but could possibly fold upon target binding [23]. This domain could thus influence the stability and/or function of ObgE* and thereby contribute to ObgE*-mediated toxicity. However, other explanations for the role of the C-terminal domain in ObgE* toxicity are possible (see below).

Mutations that abolish nucleotide binding neutralize ObgE*-mediated toxicity completely. To assess which nucleotide binding state is responsible for toxicity, we measured survival upon expression of rationally selected ObgE* mutant proteins with altered binding affinities. The effects of the mutations on the binding affinity for the different nucleotides suggest that ObgE* needs to bind GTP to exert its toxic effect (Figure 2). Moreover, we also selected spontaneous mutations that counteract ObgE* toxicity and confirmed that they have a similar effect on GTP binding (Table 3). These two independent and complementary experiments thus both point towards an important role for GTP binding in ObgE* activity. We conclude that, besides the presence of the N-terminal domain, GTP binding is also a prerequisite for ObgE*-mediated cell death. 

Since the N-terminal domain of ObgE* is essential for toxicity, we would expect that also mutations in this part of the protein could neutralize toxicity, for example by blocking binding to a downstream effector protein. However, no such mutations were isolated when looking for compensatory mutations in obgE*. We therefore suspect that our screen was not saturating and that still other mutations could be isolated that neutralize toxicity.

Interestingly, it was shown previously that deletion of the C-terminus decreases GTP binding affinity 10-fold, while having only very little effect on GDP or ppGpp binding [9]. The increased affinity for GTP in the presence of the C-terminal domain could explain the C-terminus’ capability to modestly increase toxicity. It is therefore possible that the C-terminus has no structural role in toxicity but rather decreases survival upon ObgE* expression by increasing GTP binding. 

An alternative explanation for the observed data is that not GTP binding, but rather GTP hydrolysis triggers ObgE*-mediated cell death. However, we deem this explanation very unlikely since GTP hydrolysis by the ObgE protein was shown to occur very slowly [2,5]. Moreover, it was shown previously that ObgE’s GTP hydrolysis rate is lowered by the introduction of the D246G mutation [7]. This mutation does not have any effect on ObgE*-mediated cell death, further indicating that toxicity is not linked to GTP hydrolysis.

As a GTPase, ObgE most likely adopts different conformations depending on the nucleotide-bound state [1,8,9,10]. However, these different conformations have not yet been observed experimentally [8,9]. The 4-amino-acid deletion of residues LLEL at position 153–156 that neutralizes ObgE*-mediated toxicity is located at the interface between the N-terminal and G domains. This region corresponds to the C-terminal residues of the last β-strand in the β-barrel subdomain of the Obg domain of the ObgE structure, just adjacent to the linker region connecting the Obg domain with the G domain [9]. A deletion in such a β-strand might seem deleterious to the fold at first sight. However, we noted that the deleted LLEL region is followed by a rather similar MLLA motif in the linker region (Figure 3). We therefore hypothesize that in the deletion mutant, the deleted β-strand region could be replaced by this particular part of the linker between the N and G domains, in effect resulting in a shortened linker region. This shortened linker could influence the movement of the N and G domains relative to each other and thereby affect the conformation adopted by ObgE* [12]. Due to GTP-bound ObgE* triggering a drop in viability, we suspect that the corresponding conformation of the N-terminal domain confers toxicity. When residues 153 to 156 are deleted from the protein, ObgE* is probably prevented from adopting this harmful conformation.

Taken together, this study shows how altered nucleotide binding can affect the *in vivo* activity of ObgE*. We have shown that the toxic effect of the mutant ObgE* protein depends on GTP binding, leading us to hypothesize that certain functions of the wild-type ObgE protein are likewise activated upon binding of GTP. The correct execution of this GTP-dependent functionality is most likely corrupted by the mutation present in ObgE*, leading to cell death. On the other hand, not all functions of ObgE depend on GTP binding. Most strikingly, we here show that several mutant proteins that are unable to bind nucleotides are still able to support viability (Figure S3). These results indicate that the essential function of ObgE does not depend on nucleotide binding and/or GTP hydrolysis. Moreover, it was recently shown that the induction of the antibiotic-tolerant persistent state by ObgE is not linked to GTP binding, but more likely depends on GDP and/or ppGpp [7]. It is currently unknown how GTP binding affects the conformation of ObgE. Although crystal structures of Obg proteins from several organisms are available in the apo-form or bound to GDP or ppGpp [8,9,10], the structure of Obg-GTP has not been resolved yet. However, data presented here indicate that GTP binding to ObgE indeed induces conformational changes that are transmitted to the N-terminal domain to influence protein activity. Further research is necessary to reveal how Obg functionality is controlled by nucleotide binding and which conformation is adopted by Obg when it binds GTP. 

## 4. Materials and Methods

### 4.1. Strains, Plasmids, and Growth Conditions

*E. coli* BW25113 was used for all experiments unless mentioned otherwise. Testing the influence of (p)ppGpp on toxicity was performed with *E. coli* BW25113 Δ*relA* Δ*spoT* [13]. pBAD33Gm was used to express all ObgE and ObgE* mutant proteins for *in vivo* experiments unless mentioned otherwise [19]. The *obgE* or obgE* fragments encoding the G domain, the N-terminal and G domains, and the G and C-terminal domains were amplified from pBAD/His A-*obgE* or pBAD/His A-obgE* [21] by primer pairs SPI12297/12298, SPI12299/12298, and SPI12297/10603, respectively (Table 4). Amplified fragments were digested with EcoRI and HindIII and cloned into pBAD33Gm [19]. pBAD33Gm-obgE*-*venus* was constructed by amplification of obgE*-*venus* from pBAD/His A-obgE*-*venus* [24] with primer pairs SPI10908/10909. Amplified fragments were digested with KpnI and HindIII and ligated into pBAD33Gm. obgE* mutant alleles were constructed by introducing site-specific point mutations into pBAD33Gm-obgE* [19] and/or pET28a-*obgE* by PCR amplification with mismatch primer pairs SPI12307/12308 for G166V, SPI11077/11078 for T174I, SPI11079/11080 for T193A, SPI11083/11084 for D246G, SPI12309/12310 for S270I, SPI12311/12312 for N283I, SPI12313/12314 for D286Y, SPI11085/11086 for D286L, and SPI12315/12316 for I313N. Unique codons for amino acids 153–156 were introduced into pBAD33Gm-obgE*-*venus* by amplification with mismatch primer pair SPI12303/12304. Spontaneous compensatory mutations were introduced into pET28a-*obgE* by amplification with mismatch primer pairs SPI12663/12664, SPI12764/12765, and SPI12659/12660 for mutations Δ153–156, H234R, and E265K, respectively. Prior to the transformation of PCR products to *E. coli* BW25113, the template DNA was digested with DpnI. Correct construction of all described plasmids was verified by Sanger sequencing. Overnight cultures containing a pBAD33Gm plasmid were diluted 100 times in lysogeny broth (LB) containing gentamicin (25 μg/mL) and incubated at 37 °C with continuous shaking at 200 rpm. When the OD595 nm reached 0.4, expression from pBAD33Gm was induced by the addition of 0.2% arabinose (w/v).

### 4.2. Survival Assay

To measure survival, cellular viability upon overexpression of ObgE or ObgE* was determined. Two hours after induction with arabinose, cultures were serially diluted in 10 mM MgSO_4_ and plated out on selective LB medium containing 1.5% agar. After overnight incubation at 37 °C, colonies were counted and CFUs were calculated. 

### 4.3. Identification of Non-Toxic ObgE* Alleles

Overnight cultures of *E. coli* BW25113 pBAD33Gm-obgE*-*venus* were plated on selective medium containing 0.2% arabinose. After overnight incubation, colonies that displayed yellow fluorescence were transferred to fresh selective plates with 0.2% arabinose. Plasmids from strains that were able to grow and retained fluorescence were purified and transformed to a fresh *E. coli* BW25113 background. Transformed *E. coli* were first selected on medium without arabinose and were then transferred to selective medium with 0.2% arabinose. Plasmids that had lost the ability to confer toxicity in the presence of arabinose were selected for further analysis. A total of 20 of such plasmids were sent for Sanger sequencing.

### 4.4. Identification of ObgE Mutant Alleles That Can Support Viability

After transformation of *E. coli* BW25113 with pBAD33Gm-*obgE*, the chromosomal *obgE* gene was deleted by homologous recombination according to the protocol developed by Datsenko and Wanner [25] using primers SPI11765 and SPI11766 (Table 4). Deletion of chromosomal *obgE* was performed in the presence of arabinose, the inducer of expression from the pBAD33Gm plasmid. Afterwards the chromosomal *obgE* deletion was transferred to *E. coli* BW25113 containing a pBAD33Gm plasmid encoding a mutant *obgE* allele by P1 transduction in the presence of arabinose. Successful deletions were confirmed by PCR. Constructed strains were grown overnight in the presence of arabinose and serial dilutions of these cultures were spotted onto LB agar medium with arabinose or glucose.

### 4.5. Expression, Quantification, and Purification of ObgE Proteins

All pET28a vectors, carrying an open reading frame coding for an N-terminally His_6_-tagged ObgE protein construct (wild type or mutant form), were transformed into *E. coli* BL21 (DE3) pLysS T_1_^R^ cells. Cells were grown at 37 °C in 1 L cultures of Terrific Broth (TB) medium containing 25 µg/mL kanamycin. When an OD_600nm_ of 0.8 was reached, cultures were induced with 1 mM of isopropyl β-D-1-thiogalactopyranoside (IPTG), after which they were further incubated overnight at 25 °C. In case of the ObgE_H234R_ and ObgE_Δ153–156_ mutant proteins, a chaperone-induced expression protocol was used. The temperature was lowered to 18 °C when the cultures reached an OD_600nm_ of 1. After 90 min, 0.01 M benzylalcohol was added. After growing the cultures for another 30 min, they were induced with 1 mM IPTG and further incubated overnight at 18 °C. Proteins were purified as previously described [9]. In short, all proteins were purified in two steps using Ni^2+^-NTA affinity chromatography and size exclusion chromatography. During the purification, the proteins were made nucleotide-free through an alkaline phosphatase treatment. In case of the ObgE_H234R_ and ObgE_Δ153–156_ mutants, an extra chaperone wash step was performed during the Ni^2+^-NTA affinity chromatography by using a buffer containing 5 mM ATP. 

To quantify the expression level of ObgE mutant proteins, a Western blot with anti-ObgE antibody was performed. *E. coli* was grown until an OD_600nm_ of 0.2 was reached after which expression from pBAD33Gm was induced with 0.2% arabinose for 2 h. Cells were harvested and Western blot was performed as described previously [13].

### 4.6. Isothermal Titration Calorimetry

Isothermal titration calorimetry (ITC) was used to determine the equilibrium dissociation constants (K_D_) and binding stoichiometries (n) of the different ObgE proteins (wild type or mutant) for GDP, ppGpp, and GTPγS. The ITC experiments were performed at 25 °C on a MicroCal PEAQ-ITC isothermal titration calorimeter (Malvern Panalytical) using a reference power of 10 µcal/s. Depending on the affinity, a protein concentration between 50 and 90 µM and a ligand concentration between 525 µM and 4.5 mM were used in the cell and syringe, respectively. All measurements were performed in a buffer containing 20 mM HEPES (pH 7.5), 150 mM NaCl, 5 mM MgCl_2_, and 1 mM β-mercaptoethanol. During each ITC experiment, 18 injections of 2 µL were performed, preceded by a test injection of 0.5 µL. To determine the K_D_-values and binding stoichiometries, the obtained data were fit on the single binding site model provided by the MicroCal PEAQ-ITC analysis software. In case of the ObgE_T174I_ protein, which displayed low binding affinities for all tested nucleotides, only the K_D_-value could be derived from the fit, while the binding stoichiometry was fixed at 1.

## Figures and Tables

**Table 1:** Nucleotide binding affinities (equilibrium dissociation constants, K_D_ in µM) of wild-type and mutant ObgE proteins. Equilibrium dissociation constants (K_D_) (± fitting error) were determined by isothermal titration calorimetry (ITC). The three right columns give the ratios of the K_D_ values for different nucleotides. NMB, no measurable binding.

	Equilibrium Dissociation Constants			Ratios		
	K_D_(GDP)	K_D_(GTPγS)	K_D_(ppGpp)	K_D_(GDP)/K_D_(ppGpp)	K_D_(GTPγS)/K_D_(GDP)	K_D_(GTPγS)/K_D_(ppGpp)
Wildtype ^a^	0.44 ± 0.03	1.3 ± 0.1	0.63 ± 0.08	0.70	3.0	2.1
G166V ^a^	NMB	NMB	NMB	NMB	NMB	NMB
T174I	115 ± 2	160 ± 11	81 ± 3	1.4	1.4	2.0
T193A ^a^	0.53 ± 0.03	4.7 ± 0.4	0.8 ± 0.1	0.66	8.9	5.9
D246G ^a^	4.1 ± 0.4	14.8 ± 0.9	7.8 ± 1.2	0.53	3.6	1.9
S270I ^a^	0.45 ± 0.05	4.9 ± 0.5	1.14 ± 0.09	0.39	11	4.3
N283I ^a^	NMB	NMB	NMB	NMB	NMB	NMB
D286Y ^a^	NMB	NMB	NMB	NMB	NMB	NMB

^a^ [7].

**Table 2:** Spontaneous compensatory mutations that neutralize ObgE*-mediated toxicity. Reference sequence based on obgE*. AA, amino acid. Bp, base pairs. SNP, single nucleotide polymorphism.

Type	Reference Sequence	Allele	AA Change	Frequency Found
SNP	ATA	AAA	I268K	5/9
SNP	CAC	CGC	H234R	1/9
SNP	GAG	GAT	E265D	1/9
SNP	GAG	AAG	E265K	1/9
Deletion, 6 bp	GAA AGC	-	Δ_298–299_KA	1/9

**Table 3:** Nucleotide binding affinities (equilibrium dissociation constants, K_D_ in µM) of wild-type ObgE and spontaneous compensatory mutant ObgE proteins. Equilibrium dissociation constants (K_D_) (± fitting error) were determined by isothermal titration calorimetry (ITC). The three right columns give the ratios of the K_D_ values for different nucleotides. NMB, no measurable binding.

	Equilibrium Dissociation Constants			Ratios		
	K_D_(GDP)	K_D_(GTPγS)	K_D_(ppGpp)	K_D_(GDP)/K_D_(ppGpp)	K_D_(GTPγS)/K_D_(GDP)	K_D_(GTPγS)/K_D_(ppGpp)
Wildtype ^a^	0.44 ± 0.03	1.3 ± 0.1	0.63 ± 0.08	0.70	3.0	2.1
Δ153–156	0.60 ± 0.05	3.4 ± 0.5	0.64 ± 0.06	0.94	5.7	5.3
H234R	0.43 ± 0.05	3.7 ± 0.5	0.60 ± 0.06	0.72	8.6	6.2
E265K	0.25 ± 0.03	5.7 ± 0.8	0.31 ± 0.03	0.81	23	18

^a^ [7].

**Table 4:** Primers used in this study.

Name	Sequence (5′–3′)
SPI10603	AGCCAAGCTTTTAACGCTTG
SPI10908	CACCGGTACCCACCAGGAGGAATTAACCATGAAGTTTGTTGATGAAGCATCG
SPI10909	AGCCAAGCTTCGAATTCTTA
SPI11077	AAATCGATCTTTATTCGTGCGG
SPI11078	AATAAAGATCGATTTACCCGCG
SPI11079	ACCGCGCTGGTGCCAAGTCTGGGTG
SPI11080	TGGCACCAGCGCGGTAAACGGATAATC
SPI11083	CTGTTGCACCTCATCGGCATCG
SPI11084	TCGATGCCGATGAGGTGCAAC
SPI11085	AAGATCCTGCTGCTGGATAAGGT
SPI11086	CAGCAGCAGGATCTTGTTGAACA
SPI11765	GCGTAGCGCATCAGGCTGATTTGGCGTTTATCATCAGTGACATATGAATATCCTCCTTA
SPI11766	ATCGCAACCCCGCGCAGGCGAATGATTTACGGAGAATAAAGTGTAGGCTGGAGCTGCTTC
SPI12297	TAGCGAATTCGAGCTCAGGAGGAATTAACCATGCTGCTGGCTGACGTCGGTA
SPI12298	AGCCAAGCTTTTAAGCCTGCACGACCGGG
SPI12299	TAGCGAATTCGAGCTCAGGA
SPI12303	GCGAGCTGCTTCTCGAATTGATGCTGCTGGCTGACG
SPI12304	AGCAGCATCAATTCGAGAAGCAGCTCGCGCTTATCG
SPI12307	CGTCGGTATGTTGGTGATGCCAAACGCGG
SPI12308	CCGCGTTTGGCATCACCAACATACCGACG
SPI12309	GAAATATACATCCAGGATCTG
SPI12310	ATCCTGGATGTATATTTCCAG
SPI12311	TTAGTGTTCATCAAGATCGATCTG
SPI12312	ATCGATCTTGATGAACACTAACC
SPI12313	AAGATCTATCTGCTGGATAAGGT
SPI12314	CAGCAGATAGATCTTGTTGAACA
SPI12315	TATCTGAACTCTGCGGCGAG
SPI12316	CGCCGCAGAGTTCAGATAATATTT
SPI12659	TTATCAGCAAGCTGGAAAAATACAG
SPI12660	TTTTCCAGCTTGCTGATAATAATACG
SPI12663	CGAGCTGATGCTGCTGGCTGAC
SPI12664	GCAGCATCAGCTCGCGCTTATCG
SPI12764	CTTCCTGAAGCGCCTGGAACGTTGCCGCGTCCTGT
SPI12765	CGTTCCAGGCGCTTCAGGAAGCGAATGCCCAGACC

## Supplementary Materials

Supplementary materials can be found at https://www.mdpi.com/1422-0067/21/1/16/s1.

## References

[1] Vetter I.R., Wittinghofer A. (2001). The guanine nucleotide-binding switch in three dimensions. Science.

[2] Verstraeten N., Fauvart M., Versees W., Michiels J. (2011). The universally conserved prokaryotic GTPases. Microbiol. Mol. Biol. Rev..

[3] Kint C., Verstraeten N., Hofkens J., Fauvart M., Michiels J. (2014). Bacterial Obg proteins: GTPases at the nexus of protein and DNA synthesis. Crit. Rev. Microbiol..

[4] Lin B., Covalle K.L., Maddock J.R. (1999). The *Caulobacter crescentus* CgtA protein displays unusual guanine nucleotide binding and exchange properties. J. Bacteriol..

[5] Wout P., Pu K., Sullivan S.M., Reese V., Zhou S., Lin B., Maddock J.R. (2004). The *Escherichia coli* GTPase CgtA_E_ cofractionates with the 50S ribosomal subunit and interacts with SpoT. a ppGpp synthetase/hydrolase. J. Bacteriol..

[6] Persky N.S., Ferullo D.J., Cooper D.L., Moore H.R., Lovett S.T. (2009). The ObgE/CgtA GTPase influences the stringent response to amino acid starvation in *Escherichia coli*. Mol. Microbiol..

[7] Verstraeten N., Gkekas S., Kint C.I., Deckers B., Van den Bergh B., Herpels P., Louwagie E., Knapen W., Wilmaerts D., Dewachter L. (2019). Biochemical determinants of ObgE-mediated persistence. Mol. Microbiol..

[8] Buglino J., Shen V., Hakimian P., Lima C.D. (2002). Structural and biochemical analysis of the Obg GTP binding protein. Structure.

[9] Gkekas S., Singh R.K., Shkumatov A.V., Messens J., Fauvart M., Verstraeten N., Michiels J., Versees W. (2017). Structural and biochemical analysis of *Escherichia coli* ObgE. A central regulator of bacterial persistence. J. Biol. Chem..

[10] Kukimoto-Niino M., Murayama K., Inoue M., Terada T., Tame J.R., Kuramitsu S., Shirouzu M., Yokoyama S. (2004). Crystal structure of the GTP-binding protein Obg from *Thermus thermophilus* HB8. J. Mol. Biol..

[11] Lee Y., Bang W.Y., Kim S., Lazar P., Kim C.W., Bahk J.D., Lee K.W. (2010). Molecular modeling study for interaction between *Bacillus subtilis* Obg and Nucleotides. PLoS ONE.

[12] Chatterjee A., Acharjee A., Das S., Datta P.P. (2019). Deletion analyses reveal insights into the domain specific activities of an essential GTPase CgtA in *Vibrio Cholerae*. Arch. Biochem. Biophys..

[13] Verstraeten N., Knapen W.J., Kint C.I., Liebens V., Van den Bergh B., Dewachter L., Michiels J.E., Fu Q., David C.C., Fierro A.C. (2015). Obg and membrane depolarization are part of a microbial bet-hedging strategy that leads to antibiotic tolerance. Mol. Cell.

[14] Feng B., Mandava C.S., Guo Q., Wang J., Cao W., Li N., Zhang Y., Zhang Y., Wang Z., Wu J. (2014). Structural and functional insights into the mode of action of a universally conserved Obg GTPase. PLoS Biol..

[15] Caldon C.E., March P.E. (2003). Function of the universally conserved bacterial GTPases. Curr. Opin. Microbiol..

[16] Sikora A.E., Zielke R., Wegrzyn A., Wegrzyn G. (2006). DNA replication defect in the *Escherichia coli cgtA*(ts) mutant arising from reduced DnaA levels. Arch. Microbiol..

[17] Kobayashi G., Moriya S., Wada C. (2001). Deficiency of essential GTP-binding protein ObgE in *Escherichia coli* inhibits chromosome partition. Mol. Microbiol..

[18] Foti J.J., Persky N.S., Ferullo D.J., Lovett S.T. (2007). Chromosome segregation control by *Escherichia coli* ObgE GTPase. Mol. Microbiol..

[19] Dewachter L., Verstraeten N., Jennes M., Verbeelen T., Biboy J., Monteyne D., Perez-Morga D., Verstrepen K.J., Vollmer W., Fauvart M. (2017). A mutant isoform of ObgE causes cell death by interfering with cell division. Front. Microbiol..

[20] Dewachter L., Verstraeten N., Fauvart M., Michiels J. (2018). An integrative view of cell cycle control in *Escherichia Coli*. FEMS Microbiol. Rev..

[21] Dewachter L., Verstraeten N., Monteyne D., Kint C.I., Versees W., Perez-Morga D., Michiels J., Fauvart M. (2015). A single-amino-acid substitution in Obg activates a new programmed cell death pathway in *Escherichia coli*. MBio.

[22] Levy D.D., Cebula T.A. (2001). Fidelity of replication of repetitive DNA in *mutS* and repair proficient *Escherichia coli*. Mutat. Res..

[23] Dyson H.J., Wright P.E. (2005). Intrinsically unstructured proteins and their functions. Nat. Rev. Mol. Cell Biol..

[24] Dewachter L., Herpels P., Verstraeten N., Fauvart M., Michiels J. (2016). Reactive oxygen species do not contribute to ObgE*-mediated programmed cell death. Sci. Rep..

[25] Datsenko K.A., Wanner B.L. (2000). One-step inactivation of chromosomal genes in *Escherichia coli* K-12 using PCR products. Proc. Natl. Acad. Sci. USA.

