# Supplementary material for: GTP Binding Is Necessary for the Activation of a Toxic Mutant Isoform of the Essential GTPase ObgE
Source: Int J Mol Sci. 2019 Dec 18;21(1):16. doi: 10.3390/ijms21010016 (PMC6982127; doi:10.3390/ijms21010016)
Supplement: Supplementary file 1 [file ijms-21-00016-s001.pdf]

Supplementary

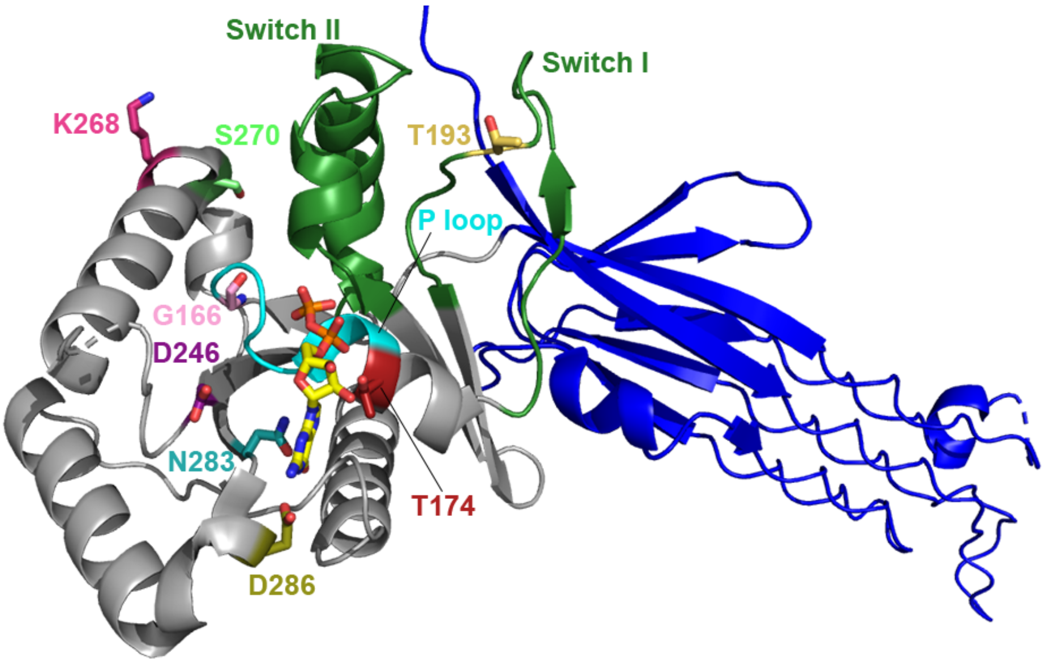

Figure S1. Crystal structure of C-terminally truncated ObgE displaying previously identified mutations that affect nucleotide binding. The N-terminal Obg domain is highlighted in blue, while the G domain is indicated in grey. The switch I and switch II loops of the G domain are colored dark green, whereas the P-loop is shown in cyan. The GDP molecule bound to the active site is displayed as sticks with the carbon atoms colored yellow. The K268 residue that is mutated in ObgE* is highlighted in dark pink. The residues that affect nucleotide binding when mutated are indicated in different colors.

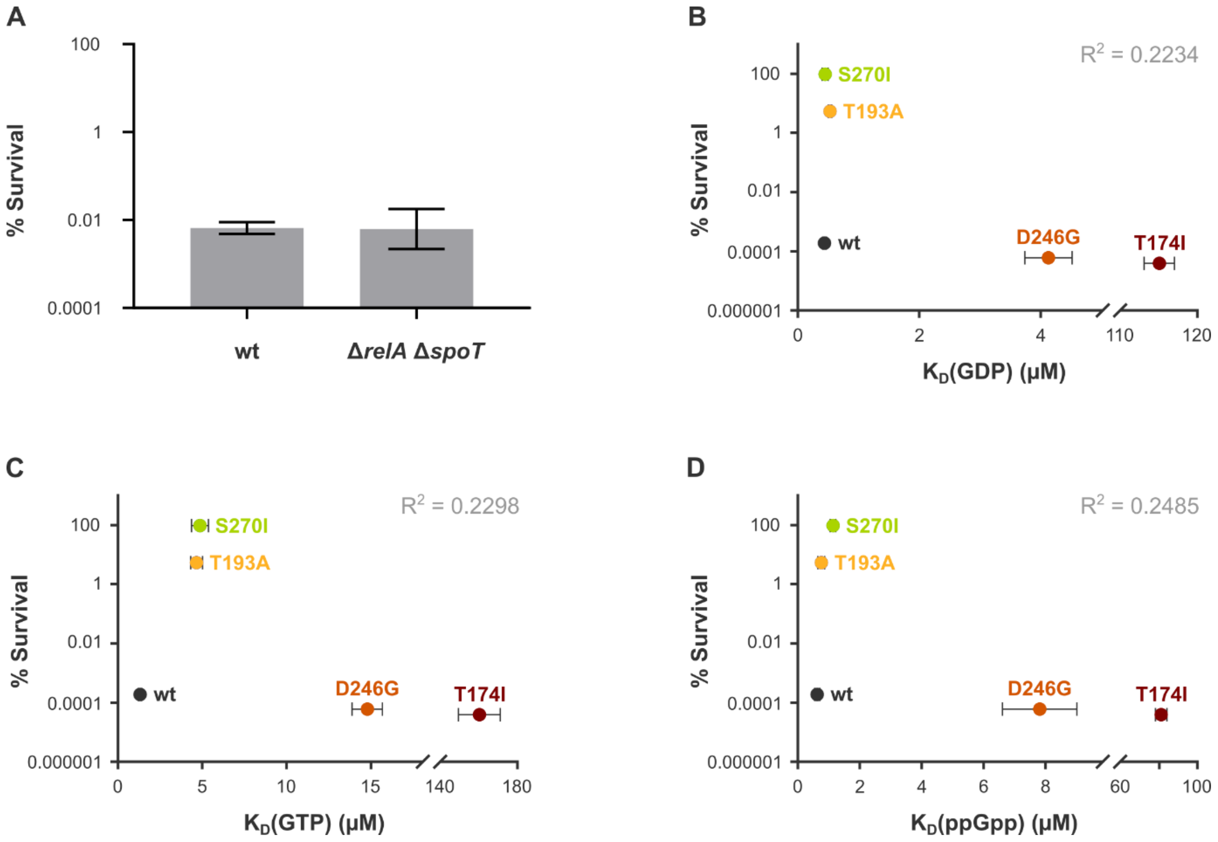

Figure S2. The influence of nucleotide binding on ObgE* toxicity. **A**) Toxicity upon expression of *obgE** in a wild-type and Δ*relA* Δ*spoT* background that is devoid of (p)ppGpp. Survival was determined by dividing the number of CFUs per ml obtained after expression of *obgE** by the number of CFUs per ml after expression of wilt-type *obgE* from pBAD/His A. Data are represented as averages ± SEM, $n \geq 3$ ($p = 0.9566$). Binding affinity for **B**) GDP, **C**) GTP and **D**) ppGpp was measured for ObgE*, ObgE*$_{T174I}$, ObgE*$_{T193A}$, ObgE*$_{D246G}$ and ObgE*$_{S270I}$ after expression from pBAD33Gm. Survival does not correlate well with any of these binding affinities. Survival data (y-axis) are represented as averages ± SEM, $n \geq 3$, binding affinities (x-axis) are displayed ± fitting error. R^2, squared Pearson correlation coefficient.

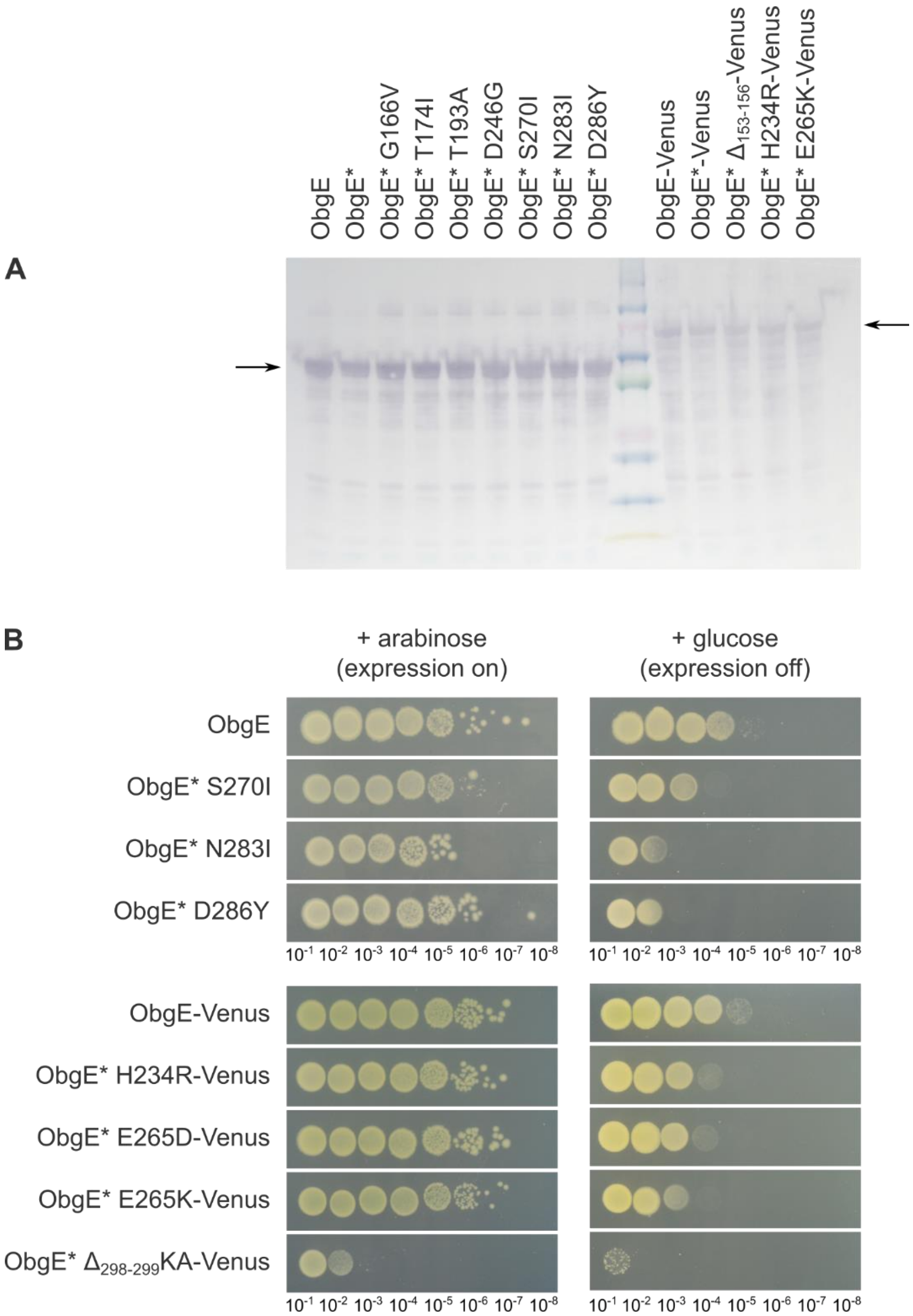

Figure S3. Selected *obgE** mutant alleles are present at similar concentrations and are correctly folded. **A**) Western blot analysis using an anti-ObgE antibody shows that mutant proteins are expressed at similar expression levels and do not show a higher level of degradation than the wildtype ObgE and ObgE* proteins. Arrows indicate bands corresponding to ObgE (and ObgE mutant proteins) and ObgE-Venus (and ObgE-Venus mutant proteins). **B**) Viability of *E. coli* BW25113 Δ*obgE* with pBAD33Gm plasmids that encode the indicated *obgE* alleles was tested. Dilution series of cultures grown overnight in the presence of arabinose were spotted on plates containing arabinose (induction of expression from P_{BAD}) or glucose (repression of expression from P_{BAD}).

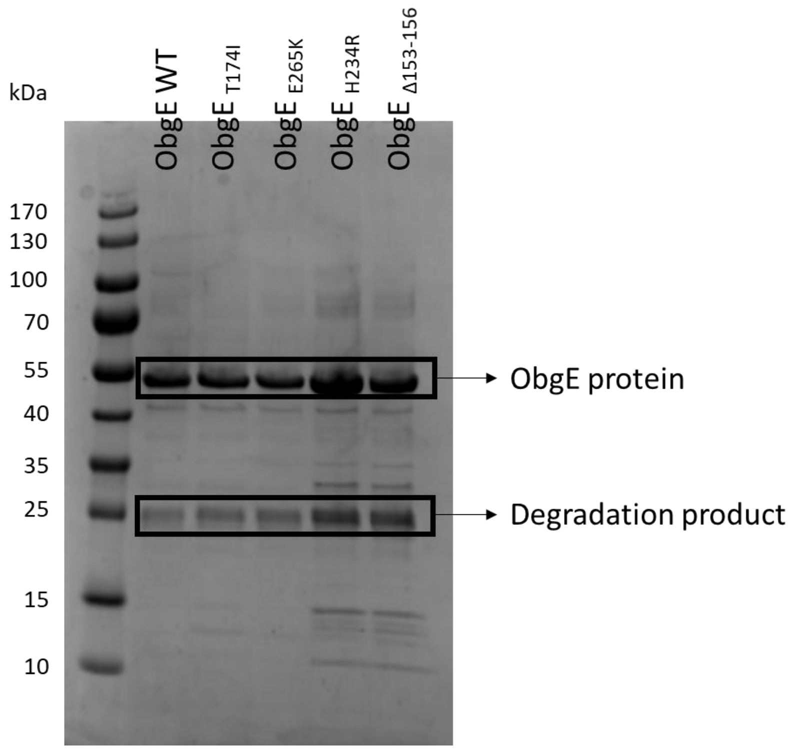

Figure S4. SDS-PAGE after purification of the $ObgE_{T174I}$, $ObgE_{E265K}$, $ObgE_{H234R}$ and $ObgE_{\Delta 153\text{-}156}$ mutant proteins. Purified wild-type ObgE protein was included as a reference. As previously observed [7], a degradation product of the ObgE protein is visible around 25 kDa, which is more pronounced for the $ObgE_{H234R}$ and $ObgE_{\Delta 153\text{-}156}$ mutants. This explains the lower binding stoichiometries that were determined for these two proteins during the ITC measurements (Figure S4).

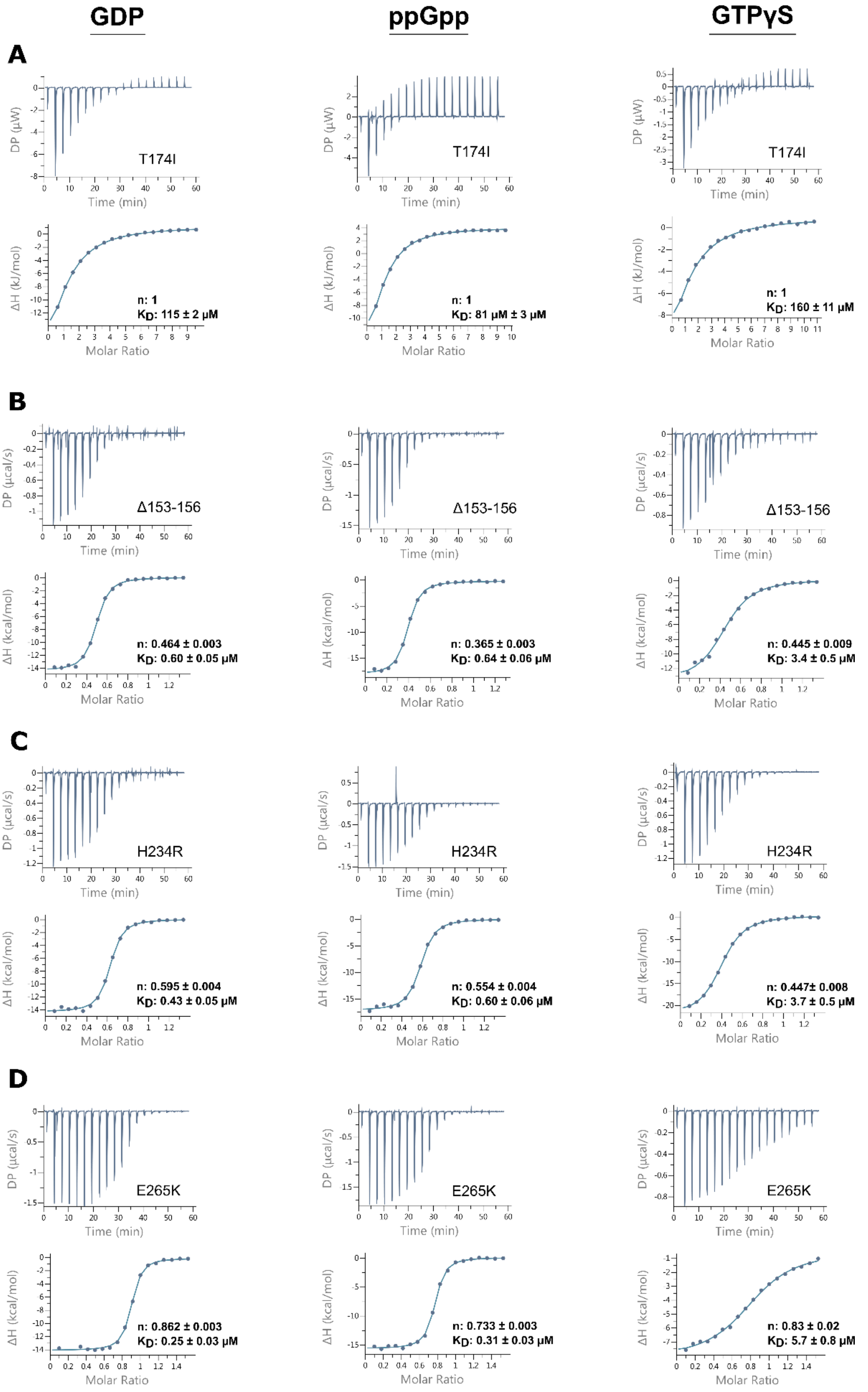

Figure S5. Isothermal titration calorimetry (ITC) experiments to determine equilibrium dissociation constants (K_D) and binding stoichiometries (n) of the different ObgE proteins (wild type or mutant) for GDP, ppGpp and GTPγS. **A**) $ObgE_{T174I}$; **B**) $ObgE_{E265K}$; **C**) $ObgE_{H234R}$; **D**) $ObgE_{\Delta 153–156}$.
